# Pilot report: objective quantification of trabecular meshwork pigmentation using densitometry and the NIDEK GS-1 gonioscope in glaucoma patients

**DOI:** 10.3389/fopht.2023.1322178

**Published:** 2024-01-23

**Authors:** Daniel Laroche, Aaron Brown, Jose Sinon, Alexander Martin, Chester Ng, Sohail Sakkari

**Affiliations:** ^1^ Department of Ophthalmology, New York Eye and Ear Infirmary, Icahn School of Medicine of Mount Sinai, New York, NY, United States; ^2^ Department of Ophthalmology, Advanced Eyecare of New York, New York, NY, United States; ^3^ Department of Ophthalmology, Downstate Medical Center, New York, NY, United States; ^4^ Department of Ophthalmology, Northwell Health, New York, NY, United States

**Keywords:** pigmentary glaucoma, biomarkers, densitometry, trabecular meshwork pigmentation, pigment dispersion glaucoma, NIDEK GS-1

## Abstract

In this case series, we present a methodology for a proof of principle for the development of a unique biomarker for pigmentary glaucoma to detect progression before nerve fiber layer loss. Out of the five patients in this case series, one was excluded because of an outlier due to pseudoexfoliation syndrome with excessively dense pigmentation of the trabecular meshwork. The remaining patients displayed a decreased visual field loss with increased superior to inferior trabecular meshwork ratios. This methodology, though limited due to small sample size, shows that in a limited number of patients, visual field loss is positively correlated with increased superior to inferior trabecular meshwork ratios. The next steps would be to look at patients without glaucoma and patients with pigmentary glaucoma, along with complete inter-eye comparisons for patients with unilateral exfoliation syndrome to act as controls. To our knowledge, this is a novel methodology, and if the pattern holds, it can act as proof of principle for the development of a novel early biomarker for pigmentary glaucoma to improve early intervention and delay vision loss.

## Introduction

Glaucoma is a group of eye diseases caused by irreversible vision loss due to retinal ganglion cell loss, optic nerve damage, and associated visual field loss. The attendant loss of retinal ganglion cell axons manifests clinically with increased cupping of the optic disc (1). The mechanism of optic nerve damage (retinal ganglion cell layer) is widely regarded as attributable to increased intraocular pressure (IOP). This increased pressure is often due to resistance to aqueous outflow via the trabecular meshwork (1). In open-angle glaucoma, the trabecular meshwork can be blocked by pigment creating an imbalance in the production and clearance of aqueous humor in the anterior chamber (2). Aqueous humor is produced by the ciliary body and mostly cleared through the trabecular meshwork. Many glaucoma cases are caused by impaired flow through the trabecular meshwork. Some treatments for glaucoma target the functionality of the trabecular meshwork with eye drops, laser irradiation, or surgical intervention (1, 2).

Pigment dispersal mechanisms are a leading cause of glaucoma, especially in patients of color (3). However, there is limited data available on the quantification of pigment in the trabecular meshwork. In one study, the Image J software was used to quantify pigment in the trabecular meshwork of three groups of patients: normal, primary open-angle glaucoma (POAG), and exfoliation glaucoma/pigmentary glaucoma (PXFG/PDG). The authors found no significant difference in normal vs. POAG but a significant one in normal vs. PXFG/PDG. They concluded that Image J was a useful tool in quantifying pigment in the trabecular meshwork (4). In another article, trabecular meshwork pigmentation in Pigment Dispersion Syndrome (PDS) patients was found to attenuate in time after reverse pupillary block was resolved (5).

We hypothesize that the accumulation of pigment in the trabecular meshwork can be quantified and thus correlated with glaucoma, elevated IOP, and visual field loss. Here, we report a case series of how pigmentation quantification of the trabecular meshwork can be used to detect glaucoma before nerve fiber layer loss and visual field loss in patients with glaucoma.

## Methods

This study was approved by the New York Eye and Ear Infirmary at Mount Sinai IRB (HS #: STUDY-21-01736). The research proceeded according to the Declaration of Helsinki with respect to scientific research. The data were collected at the offices of Advanced Eyecare of New York. Informed consent was obtained from all participants following an explanation of the possible risks.

The NIDEK GS-1 gonioscope documents the iridocorneal angle in real-color photographs and stores them in the device. Prior to imaging, the patient was administered topical anesthetic drops before being positioned in front of the machine’s prism. This prism was covered with a lubricating gel and arranged in contact with the cornea. The GS-1 gonioscope will automatically indicate if the prism needs to be adjusted to best focus on the angle structures. When the ideal focus is achieved, the device begins to capture multiple images circumferentially at different focus depths within each of the 16 sectors. The imageas acquired have a capture area per image approximately 2.36 mm (circumference direction) x 2mm (diameter direction) with working distance of 1.5mm.

The GS-1 captured the 360° horizontally concatenated images of the ICA included in this report. The study population included suspected glaucoma patients and patients with mild, moderate, and severe glaucoma, as defined by the Hodapp–Parrish–Anderson criteria. Exclusion criteria included any history of intraocular surgery, pseudoexfoliation syndrome, traditional pigment dispersion syndrome with concave iris, and transillumination present.

To objectively quantify pigment in the trabecular meshwork, densitometric analysis was performed for each image. Readings were obtained for 66 × 66 pixel boxes centered directly over the trabecular meshwork on the “I” (Inferior) and “S” (Superior) markings in each image using ImageJ software. Output data included mean, minimum, maximum, and mode. The smaller the number the darker the pixels in the reading, corresponding to the tissue with more pigment accumulation. This method provided relative, but not absolute measurements of the darkness of the angle. To normalize these measurements within patients, a ratio was created for superior to inferior mean pixel density. This ratio allowed for a comparison of density between subjects.

### Case 1

**Figure 1:**
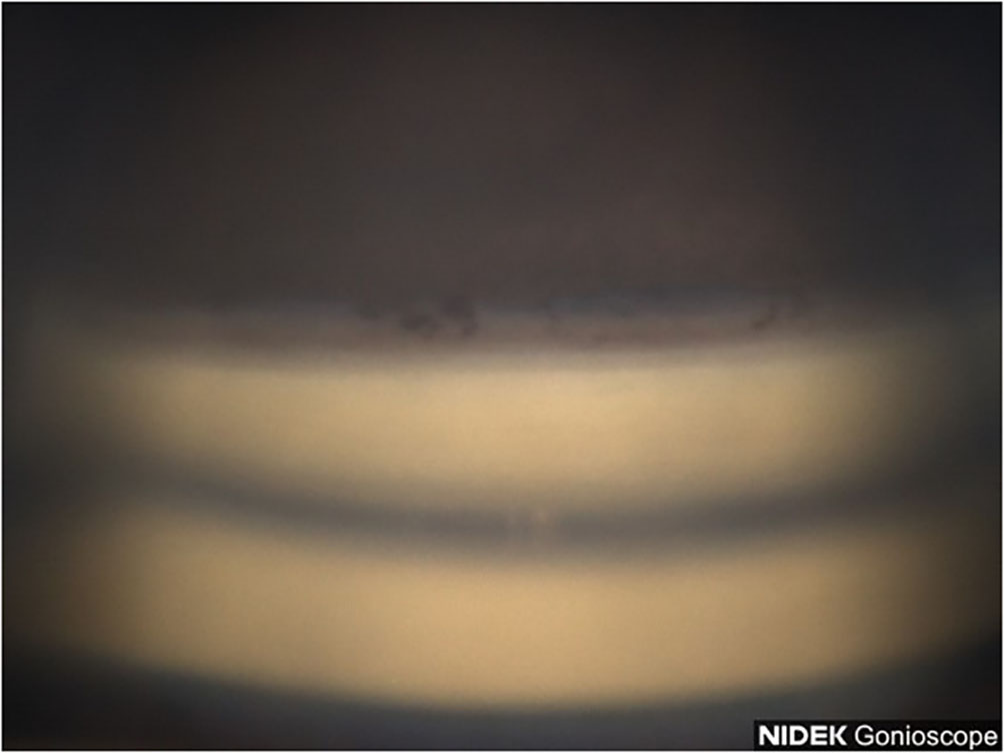
Case 1: Superior angle (inverted by mirror).

**Figure 2:**
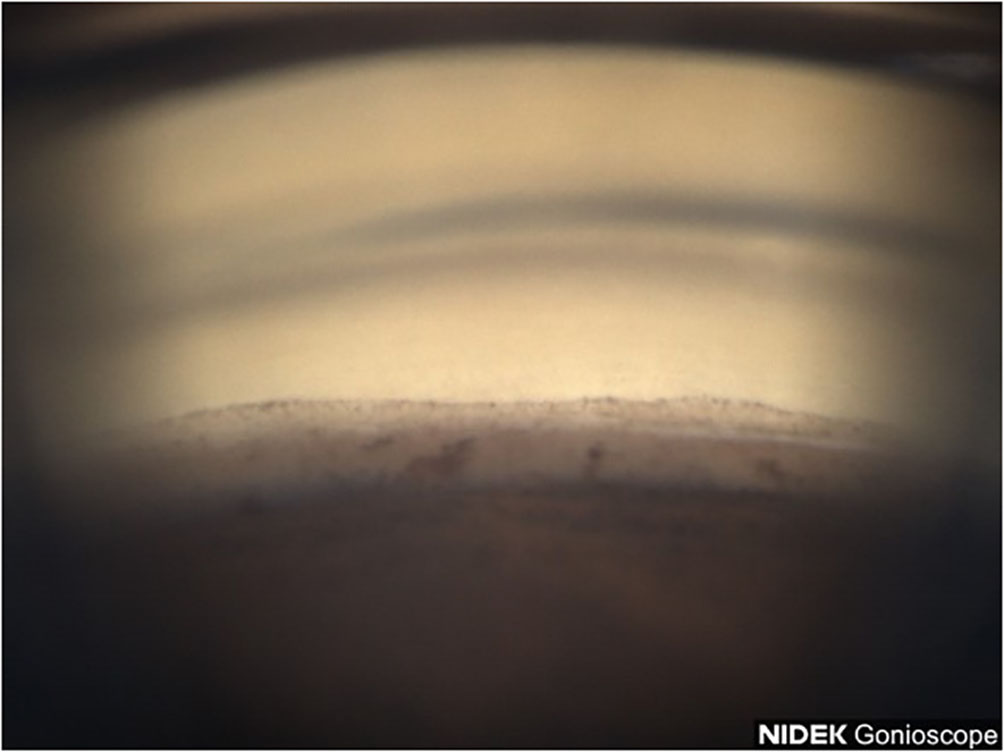
Case 1: Inferior angle (inverted by mirror).

A 77-year-old Black female patient presented with a history of mild primary open-angle glaucoma. The patient’s medical history was significant for diabetes and hypertension. She had no family history of glaucoma. She had been treated for diabetic retinopathy via focal laser in the past. Her current medications at the time included aspirin, sitagliptin, metformin, and liraglutide. She was taking latanoprost QHS for both eyes and dorzolamide/timolol BID for both eyes to control IOP. Best corrected visual acuity (BCVA) was 20/25 OU, IOP was at 12 mmHg OD and 11 mmHg OS, with a TMax of 21 OU. Slit lamp examination was remarkable for 2+ nuclear sclerotic cataracts in both eyes, cup-disc ratio (CDR) of 0.3 in both eyes, with nerve fiber layer loss in both eyes and arterial attenuation, and gonioscopic findings of Shaffer grades II–III with iris convex configuration and grade 2+ pigmentation superiorly (**Figure 1**) and pigmentation inferiorly (**Figure 2**) OD. Visual fields revealed a moderate inferior defect OD with a visual field index (VFI) of 88% and mean deviation (MD) of −9.39 dB and a mild infranasal defect OS with a VFI of 91% and MD of −4.64 dB. Optical coherence tomography (OCT) revealed average retinal nerve fiber thickness to be 95-μm OD and 100-μm OS. Goniophotos with analysis of the superior angle revealed a mean densitometry of 100.3, and the inferior angle was 90.6. The ratio of the superior to inferior angle densitometry measurements was 1.11 for the left eye with glaucoma.

### Case 2

**Figure 3:**
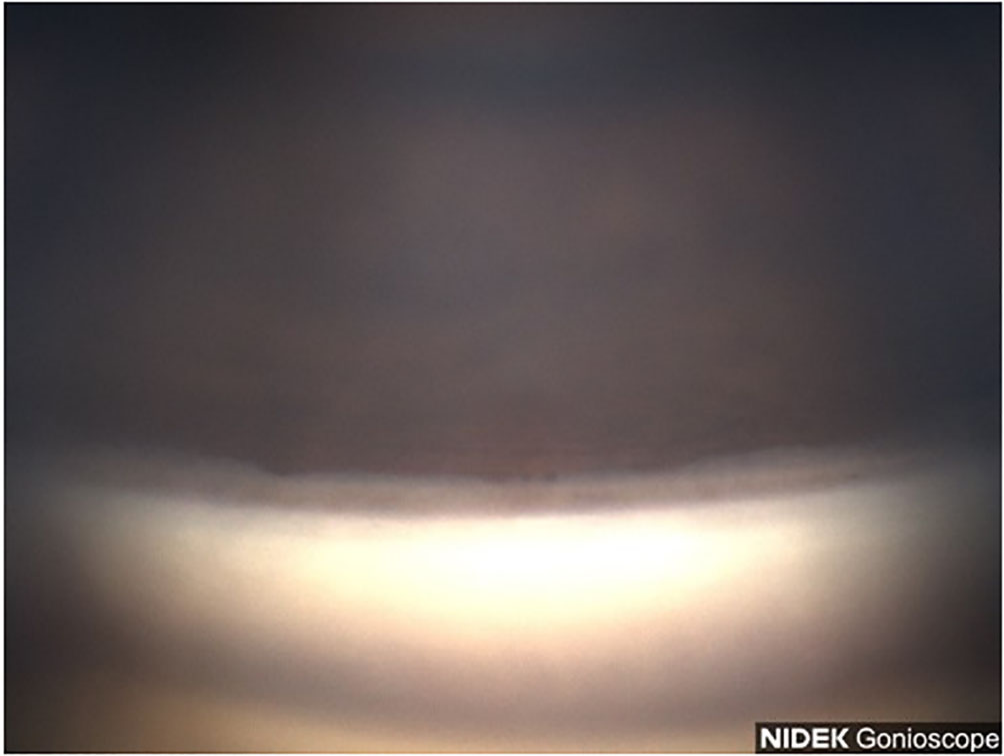
Case 2: Superior angle (inverted by mirror).

**Figure 4:**
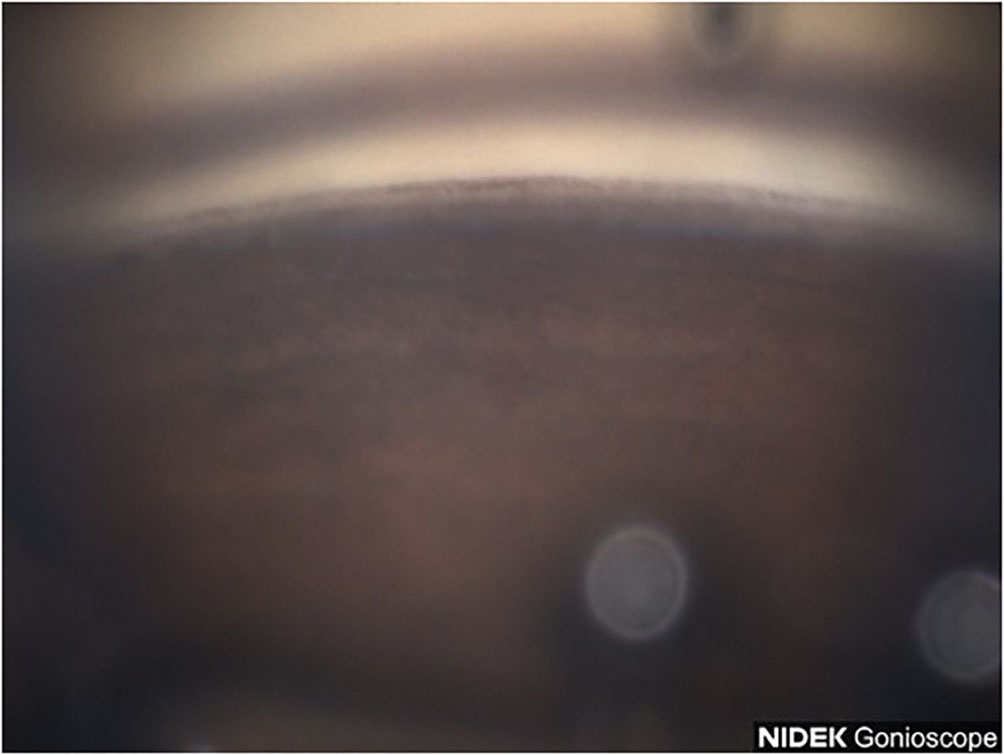
Case 2: Inferior angle (inverted by mirror).

A 53-year-old Black male patient presented with a medical history of hypertension and asthma. BCVA was 20/20 OU. IOP values were 12 mmHg OD and 11 mmHg OS. The patient had thin corneas with corneal pachymetry of 465 μm OD and 457 μm OS. The patient’s topical medication regimen included latanoprost QHS OU, dorzolamide 2% BID OS, and brimonidine 0.2% BID OS. The slit lamp exam was remarkable for mild cataracts in both eyes, cupping of the optic nerves with CDRs of 0.75 OD and 0.85 with inferior notching OS. Gonioscopy revealed open Shaffer grade IV angles OU with 2+ pigmentation in the trabecular meshwork in the superior angle (**Figure 3**) with heavier pigmentation in the inferior angle (**Figure 4**). The patient’s visual field examination revealed a VFI of 99% OD and 91% OS, and MD of −0.30 OD and −2.95 OS. OCT findings revealed an average retinal nerve fiber thickness of 75 μm OD and 66 μm OS. Goniophotos with analysis of the superior angle revealed a mean densitometry of 162.007 and the inferior angle was 126.436. The ratio of the superior to inferior angle densitometry measurement was 1.28 for the left eye. This signifies a much higher level of pigment in the inferior angle.

### Case 3

**Figure 5:**
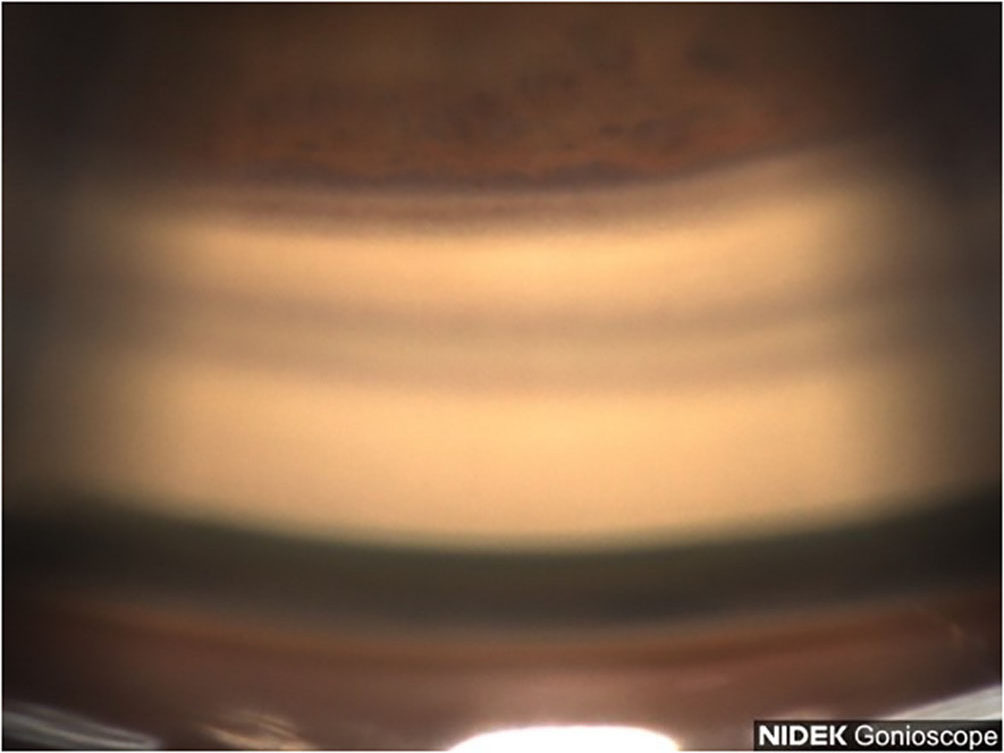
Case 3: Superior angle (inverted by mirror).

**Figure 6:**
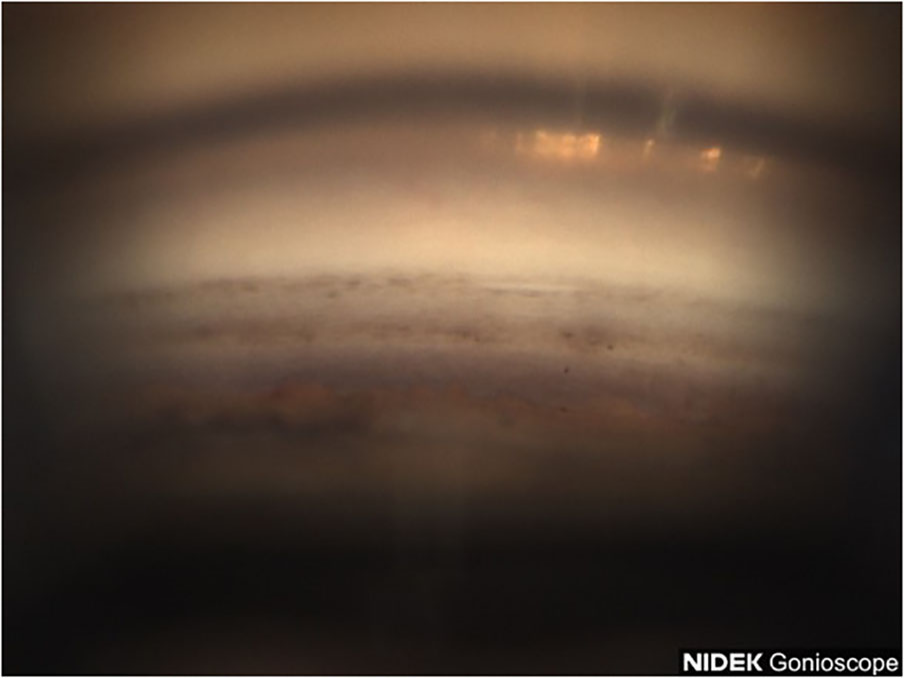
Case 3: Inferior angle (inverted by mirror).

A 52-year-old Black male patient presented as a glaucoma suspect having been told he had elevated IOP values. His medical history was remarkable for diabetes and his mother had a history of glaucoma. The patient was not taking any topical eye drops at the time. His BCVA was 20/20 OU. IOP was measured at 23 mmHg in both eyes and corneal pachymetry revealed 538 μm OD and 551 μm OS. Gonioscopy revealed Shaffer grade III angles open OU with grade 1+ pigmentation superiorly (**Figure 5**) and inferiorly (**Figure 6**) for both eyes. Anterior segment slit lamp exam was remarkable for grade 2+ nuclear sclerotic cataracts. Posterior segment exam revealed CDRs of 0.6 with mild nerve fiber loss OD and 0.7 with mild nerve fiber loss OS. OCT exam revealed global retinal nerve fiber layer (RNFL) thicknesses of 73 μm OD and 69 μm OS. Visual field exam revealed a VFI of 99% OD and MD of −0.53 dB and a VFI of 99% OS and MD of −1.83 dB. Goniophotos with analysis of the superior angle revealed a mean densitometry of 162.007 superiorly and the inferior angle was 126.436. The ratio of the superior to inferior angle densitometry measurement was 1.28 for the left eye with glaucoma. This signifies a much higher level of pigment in the inferior angle.

### Case 4

**Figure 7:**
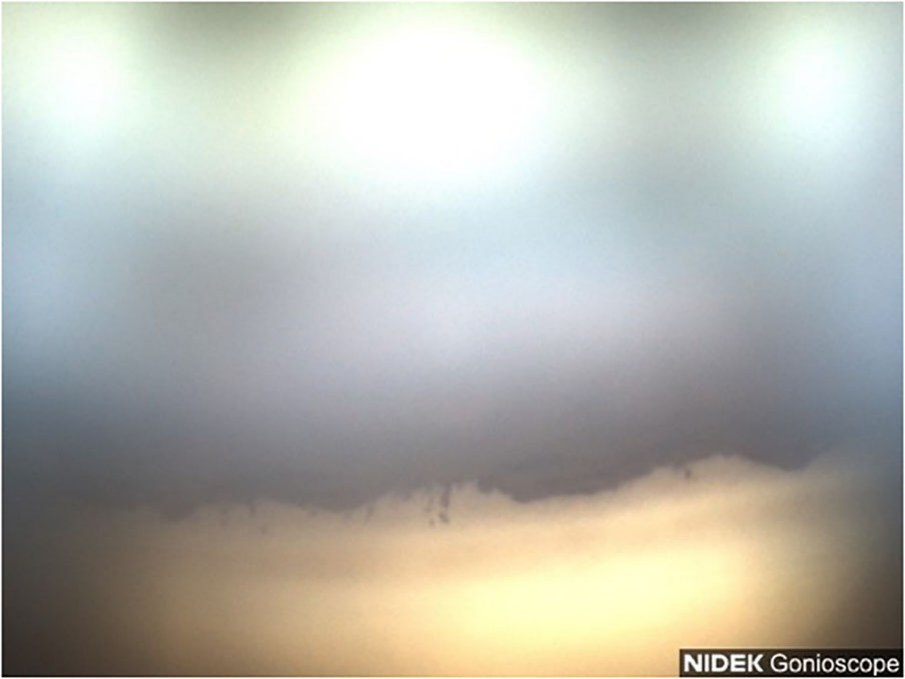
Case 4: Superior angle (inverted by mirror).

**Figure 8:**
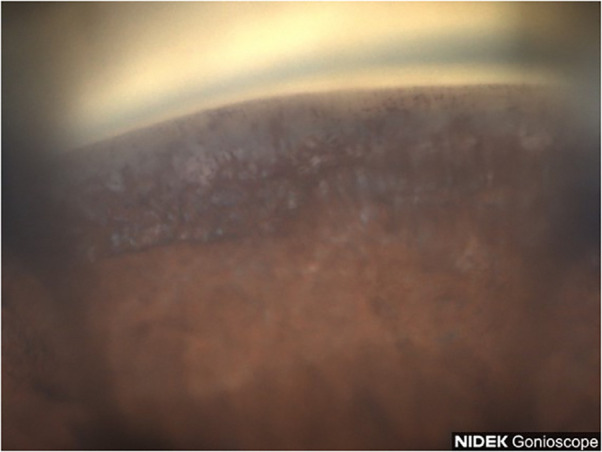
Case 4: Inferior angle (inverted by mirror).

A 57-year-old Black female patient first presented for evaluation for glaucoma. Her medical history was remarkable for diabetes, hypertension, and asthma. She did have a family history of glaucoma in her mother. Her surgical history was remarkable for retinal laser treatments related to diabetic retinopathy. The patient was not taking any topical eye drops at the time. Her BCVA was 20/40 OD and 20/50 OS. IOP values were measured at 22 mmHg OD and 20 mmHg OS. Gonioscopy revealed Shaffer grade III open angle with pigmentation of the angles in the superior quadrant (**Figure 7**) and heavier pigment in the inferior quadrant (**Figure 8**). Anterior segment slit lamp exam was remarkable for grade 1+ nuclear sclerotic cataracts in both eyes. The posterior segment slit lamp exam revealed mild non-proliferative diabetic retinopathy with exudates OD and moderate non-proliferative diabetic retinopathy with exudates OS. CDRs were seen as 0.8 OD with mild nerve fiber layer loss and 0.7 OS with suspicious cupping. OCT scan revealed an average RNFL of 75 um OD and a poor unreliable scan of the OS. Visual field examination revealed non-specific inferior defects in both eyes with a VFI of 85% OD and MD −9.37 dB and a VFI of 83% OS and MD −9.23 dB. Gonio photos with analysis of the superior angle revealed a mean densitometry of 207.37 and the inferior angle was 128.59. The ratio of the superior to inferior angle densitometry measurement was 1.61 for the right eye with glaucoma. This signifies a much higher level of pigment in the inferior angle.

### Case 5

**Figure 9:**
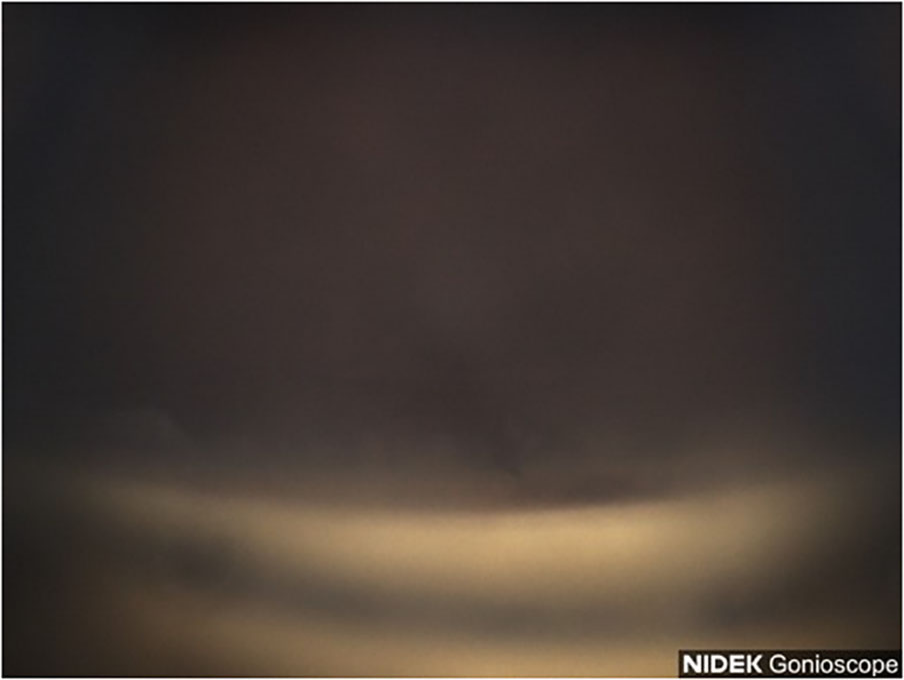
Case 5: Superior angle (inverted by mirror).

**Figure 10:**
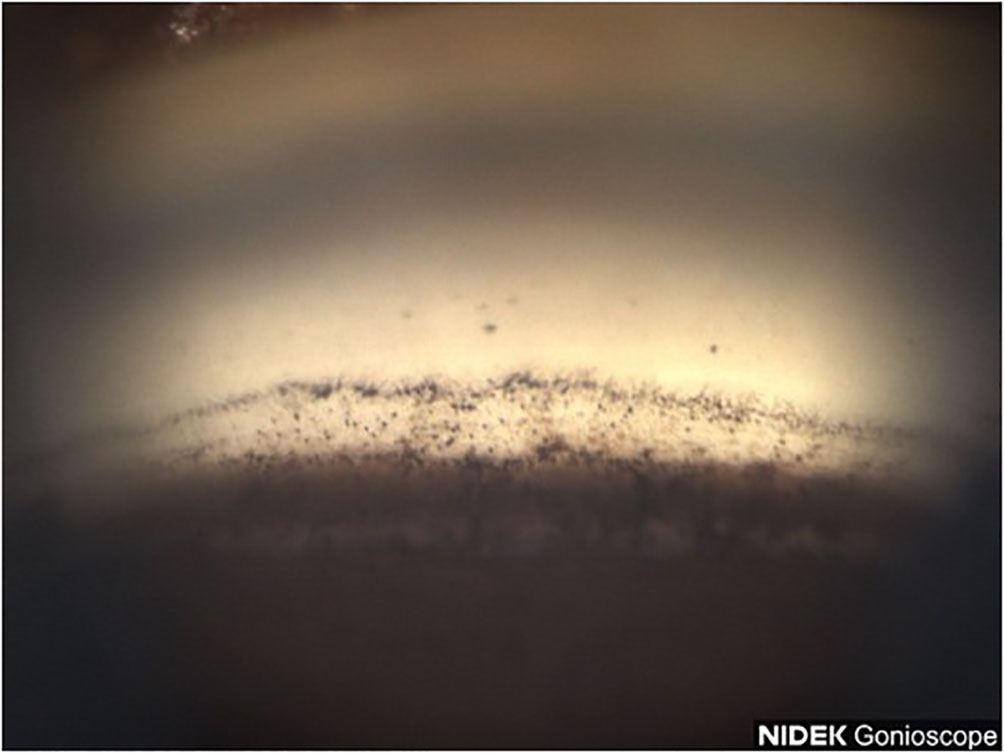
Case 5: Inferior angle (inverted by mirror).

A 52-year-old Black male reported for an initial exam complaining of blurry vision in his OS. The patient was not using any topical eye drops. The patient’s BCVA was 20/30 OD and 20/40 OS. IOP values were measured at 34 mmHg OD and 36 mmHg OS. A slit lamp examination revealed mild early nuclear sclerosis with pseudoexfoliation material present on the lens OS. Gonioscopy revealed Shaffer grade III angles in both eyes with heavy pigmentation in both the superior angle (**Figure 9**) and inferior angle (**Figure 10**). Optic nerve examination revealed glaucomatous cupping of 0.7 OD and 0.8 OS with mild nerve fiber layer defects. Visual fields revealed an early inferior arcuate defect, a VFI of 86%, and MD −12.47 dB OD and central island of vision, a VFI of 14% and MD −29.55 dB OS. Nidek GS- 1 gonioscope photos confirmed heavy grade 3+ pigmentation of the inferior trabecular meshwork of both eyes. The patient was instructed to start a regimen of latanoprost 0.005% QHS OU, dorzolamide/timolol 22.3–6.8 mg/mL BID OU, and brimonidine 0.15% TID OU. The patient was recommended to have cataract extraction with an intraocular lens placed in a sulcus and trabeculectomy OS and cataract extraction/Hydrus/Omni OD.

Goniophotos with analysis of the superior angle revealed a mean densitometry of 79.78 superiorly and the inferior angle was 58.487. The ratio of the superior to inferior angle densitometry measurements was 1.36 for the left eye with glaucoma. This signifies a much higher level of pigment in the inferior angle.

## Discussion

This case series shows how patients with heavier pigment density in the trabecular meshwork may be correlated with elevated IOP and glaucoma. The current understanding of pigment dispersion syndrome is that it is a disease that predominantly affects Caucasian men (6, 7). In practice, many patients of Latin American and African descent can be observed to have high levels of pigment in the Trabecular meshwork (TM). This can be often associated with glaucoma. The presence of pigment in the TM can obstruct the trabecular meshwork and cause elevation of intraocular pressure that can further narrow Schlemm’s canal and obstruct the collector outflow channels. The current belief is that patients of African descent may not suffer from pigmentary glaucoma at rates as high as those of Caucasians. The amount of pigment in the angle of patients with flat or convex iris insertions may have been severely underestimated. This may also be due to erroneous and inconsistent grading of TM pigment density.

Pigment dispersal syndrome is classically associated with young Caucasian adults with concave irises. This can then lead to pigmentary glaucoma. Pigmentary glaucoma is not considered a common etiology of glaucoma in populations of Black and Afro-Latino descent. This could be due to difficulty in the diagnosis of people of color. It has been reported that common exam findings such as transillumination defects are often missing in patients of color with pigment dispersion syndrome (3). For this reason, if diagnosed, patients of color usually have a later age of diagnosis than their Caucasian counterparts (8). Objective quantification of pigment, as demonstrated in this study, may address these discrepancies. This may also be able to identify the causes of ocular hypertension preceding glaucomatous nerve fiber layer loss and glaucomatous visual field loss.

Standardization techniques will be important in the future. Artificial intelligence (AI) has sparked tremendous interest recently (9). AI is based on deep learning (DL). DL has been used in ophthalmology and has been applied to fundus photographs, OCT, and visual fields. DL has achieved excellent classification performance in the detection of diabetic retinopathy and retinopathy of prematurity, the glaucoma-like disc. There may be a future role for DL in evaluating TM images and assessing pigment density in relation to glaucoma. There are also potential challenges with DL application in assessing TM, including clinical and technical challenges and new algorithms. However, future work in this area could potentially revolutionize how glaucoma workup are performed in the future. This will be of critical importance.

The superior–inferior ratio (SIR) acts as a ratio of the reading of the density of pigment picked up by the program of the superior and inferior trabecular meshwork. In our study, it ranges from 1.11 to 1.61 (**Table 1**). The highest SIR value is found in Case 4 R, which suggests that the amount of pigment in the inferior meshwork is relatively high compared to the superior portion. In contrast, the lowest SIR value is seen in Case 1 L, which suggests that the amount of pigment in the inferior portion of the trabecular meshwork is relatively low compared to the superior segment (pigment reversal sign). We chose the SIR since the superior density reading will be closest to normal, and the inferior density reading due to gravity will be the most abnormal as pigment liberation that occurs over time from irido-lenticular contact descends to the bottom of the TM.

**Table 1:** Demographic summary with superior and inferior pigment densitometry readings, ratios, and visual field mean deviation values.

	Age	Race	Sex	Medical history	BCVA	IOP	Angle	Slit lamp findings	CDR	OCT average RNFL	VF mean deviation	Pigment density		
Case number	(Years)	(A/B/H)*	(M/F)*		(Snellen)	(mmHg)	(Shaffer grade)			(μm)	(dB)	Superior	Inferior	Ratio
1	77	B	F	Diabetes, Hypertension	20/25	11	II-III	Cataracts	0.3	100	−4.64	100.3	90.6	1.11
2	53	A	M	Hypertension, Asthma	20/20	11	IV	Cataracts	0.85	66	−2.95	162.007	126.436	1.28
3	52	H	M	Diabetes	20/20	23	III	Cataracts	0.7	69	−1.83	139.26	123.66	1.13
4	57	B	F	Diabetes, Hypertension	20/40	22	III	Cataracts	0.8	75	−9.37	207.37	128.59	1.61
5	52	B	M	None	20/40	36	III	Cataracts	0.8	–	−29.55	79.789	58.487	1.36

*A, Asian; B, Black; H, Hispanic; M, Male; F, Female.

BCVA, best corrected visual acuity; IOP, intraocular pressure; CDR, cup-disc ratio; OCT, optical coherence tomography; RNFL, retinal nerve fiber layer; and VF MD, visual field mean deviation.

In addition, the visual field mean deviation (VF MD) measures the overall visual field sensitivity. In this dataset, the VF MD ranges from −29.55 dB to −1.83 dB. The most negative value is in Case 5, which indicates a significant loss of visual field. The least negative VF MD value is seen in Case 3 L, which indicates a relatively healthier visual field, though still with some degree of deterioration.

Of note is the status of Case 5 with a dramatically worse visual field due to the excessive pigment liberation both superiorly and inferiorly. The pigment density readings were both below 90 superiorly and inferiorly, making this an outlier and a possible identifier for advanced glaucoma with a dramatic worsening of the visual field, given the patient’s diagnosis of pseudoexfoliation. With the exclusion of Case 5 as an outlier, values generally show an increasing trend with increased ratios supporting a worsening visual field and a potential early biomarker for glaucoma.

**Figure 11:**
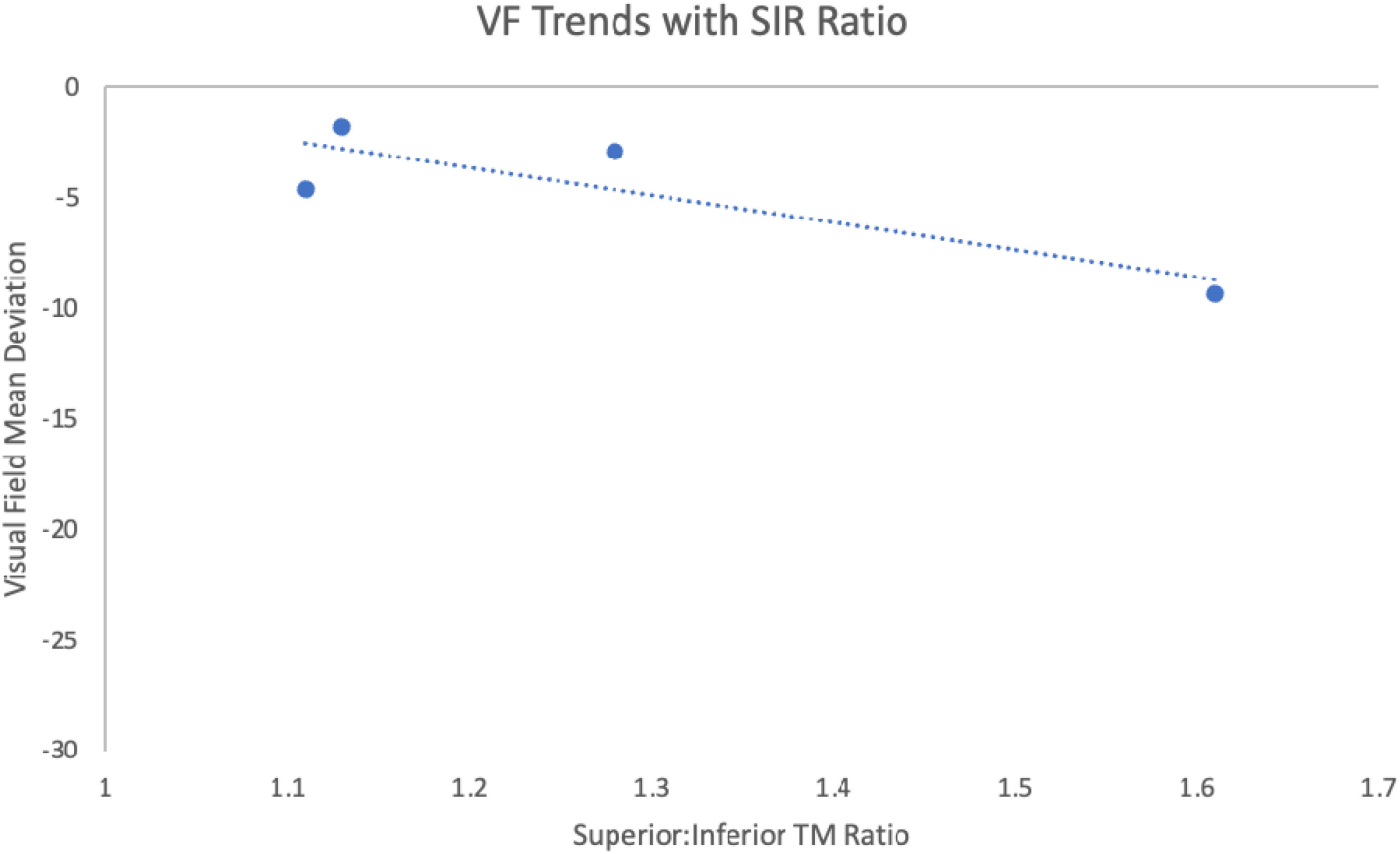
Graph: Trabecular meshwork pigment densitometry ratios with visual field trend line.

In this case series, the patients most at risk of glaucomatous field loss were those with a greater difference between the superior and inferior pigment amount; the greatest ratio corresponded to more severe glaucoma (**Figure 11**). This is a pilot report. A larger dataset is needed to further investigate this trend. If this trend holds, imaging the angle could identify patients at risk of IOP elevation and severe glaucoma before vision loss occurs. AI may also play a significant role in obtaining many trabecular meshwork images and correlating this with IOP and glaucoma. The pigment densitometry software incorporated in this analysis may play an important predicting role in screening and determining glaucomatous trabecular meshwork before glaucomatous optic nerve damage. Further research is planned and required.

## Data availability statement

The original contributions presented in the study are included in the article/supplementary material. Further inquiries can be directed to the corresponding author.

## Ethics statement

The studies involving humans were approved by New York Eye and Ear Infirmary IRB. The studies were conducted in accordance with the local legislation and institutional requirements. The participants provided their written informed consent to participate in this study. Written informed consent was obtained from the individual(s) for the publication of any potentially identifiable images or data included in this article.

## Author contributions

DL: Conceptualization, Investigation, Methodology, Project administration, Writing – original draft, Writing – review & editing. AB: Investigation, Project administration, Writing – original draft, Writing – review & editing. JS: Formal analysis, Investigation, Writing – review & editing. AM: Data curation, Formal analysis, Investigation, Methodology, Writing – review & editing. CN: Data curation, Formal analysis, Investigation, Writing – review & editing. SS: Data curation, Investigation, Writing – review & editing.

## References

[1] Chakravarti T. Assessing precision of hodapp-parrish-anderson criteria for staging early glaucomatous damage in an ocular hypertension cohort: A retrospective study. Asia Pac J Ophthalmol (Phila) Jan-Feb (2017) 6:21–7. doi: 10.1097/apo.0000000000000201 28161915

[2] Gomez Goyeneche HF Hernandez-Mendieta DP Rodriguez DA Sepulveda AI Toledo JD. Pigment dispersion syndrome progression to pigmentary glaucoma in a latin american population. J Curr Glaucoma Pract Sep-Dec (2015) 9:69–72. doi: 10.5005/jp-journals-10008-1187 PMC4779943 26997839

[3] Semple HC Ball SF. Pigmentary glaucoma in the black population. Am J Ophthalmol (1990) 109:518–22. doi: 10.1016/S0002-9394(14)70680-4 2333915

[4] Kinori M Hostovsky A Skaat A Schwartsman J Melamed S. A novel method for quantifying the amount of trabecular meshwork pigment in glaucomatous and nonglaucomatous eyes. J Glaucoma Jan (2014) 23:e13–7. doi: 10.1097/IJG.0b013e3182a0758c 24370807

[5] Križaj D. What is glaucoma. In: The organization of the retina and visual system. Salt Lake City (UT): University of Utah Health Sciences Center (2019). 21413389

[6] Laroche D Capellan P. The Aging Lens and Glaucoma in persons over 50: Why early cataract surgery/refractive lensectomy and microinvasive trabecular bypass can prevent blindness and cure elevated eye pressure. J Natl Med Assoc Aug (2021) 113:471–3. doi: 10.1016/j.jnma.2021.03.001 33781565

[7] Zhou R Tang Q Pu L Qing G. Changes of trabecular meshwork pigmentation in patients with pigment dispersion syndrome: A 15-year study. Med (Baltimore) Aug 6 (2021) 100:e26567. doi: 10.1097/md.0000000000026567 PMC8341214 34397796

[8] Scuderi G Contestabile MT Scuderi L Librando A Fenicia V Rahimi S. Pigment dispersion syndrome and pigmentary glaucoma: a review and update. Int Ophthalmol Jul (2019) 39:1651–62. doi: 10.1007/s10792-018-0938-7 29721842

[9] Ting DSW Pasquale LR Peng L Campbell JP Lee AY Raman R. Artificial intelligence and deep learning in ophthalmology. Br J Ophthalmol (2019) 103(2):167–75. doi: 10.1136/bjophthalmol-2018-313173 PMC6362807 30361278

