# Corrigendum: Pilot report: objective quantification of trabecular meshwork pigmentation using densitometry and the NIDEK GS-1 gonioscope in glaucoma patients

**DOI:** 10.3389/fopht.2024.1382567

**Published:** 2024-04-17

**Authors:** Daniel Laroche, Aaron Brown, Jose Sinon, Alexander Martin, Chester Ng, Sohail Sakkari

**Affiliations:** ^1^ Department of Ophthalmology, New York Eye and Ear Infirmary, Icahn School of Medicine of Mount Sinai, New York, NY, United States; ^2^ Department of Ophthalmology, Advanced Eyecare of New York, New York, NY, United States; ^3^ Department of Ophthalmology, Downstate Medical Center, New York, NY, United States; ^4^ Department of Ophthalmology, Northwell Health, New York, NY, United States

**Keywords:** pigmentary glaucoma, biomarkers, densitometry, trabecular meshwork pigmentation, pigment dispersion glaucoma, NIDEK GS-1

In the published article, there was an error in **Figures 6**, **7**, **8**, **9**, **10** as published. The sequence of the images was incorrect. The corrected **Figures 6**, **7**, **8**, **9**, **10** and their captions appear below.

**Figure 6:**
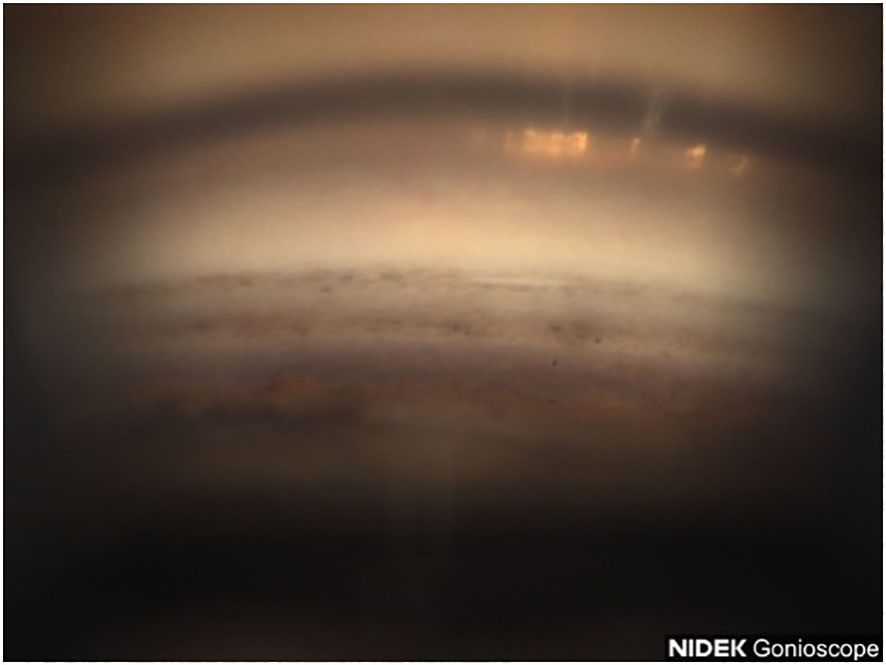
Case 3: Inferior angle (inverted by mirror).

**Figure 7:**
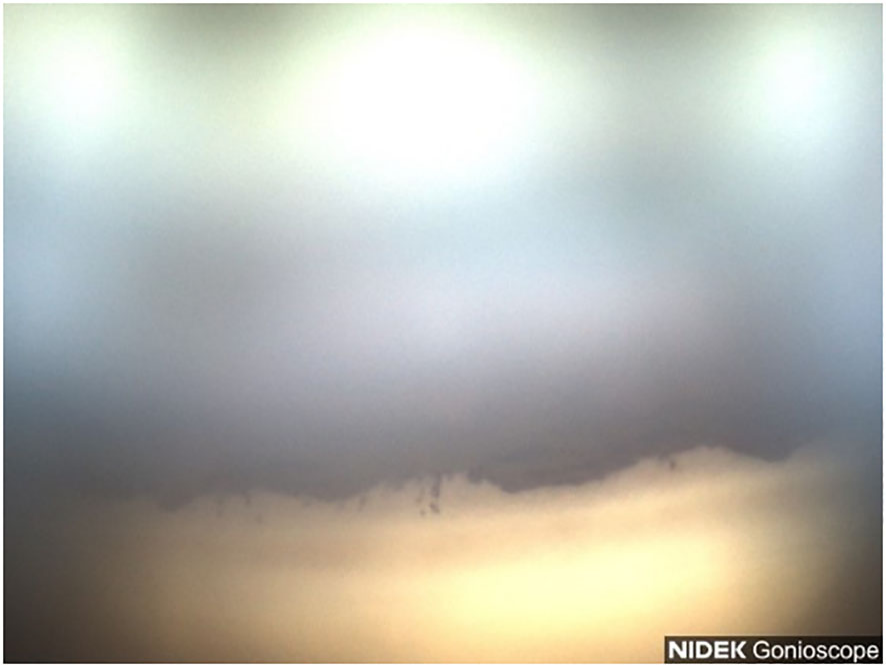
Case 4: Superior angle (inverted by mirror).

**Figure 8:**
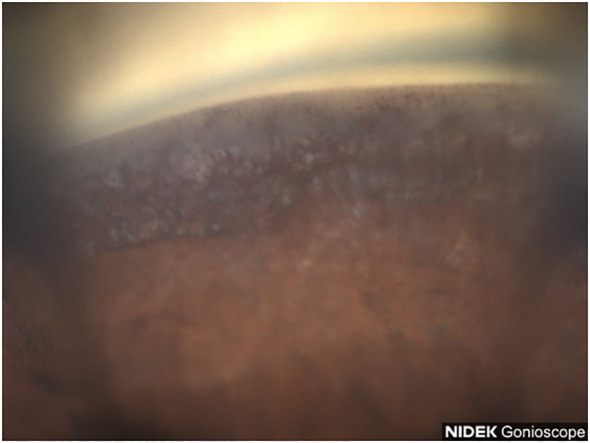
Case 4: Inferior angle (inverted by mirror).

**Figure 9:**
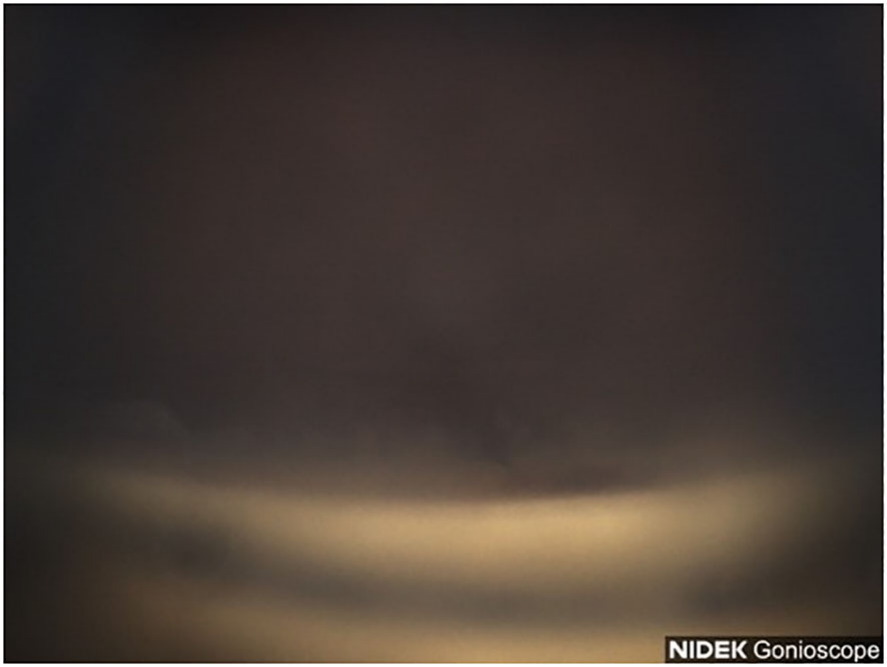
Case 5: Superior angle (inverted by mirror).

**Figure 10:**
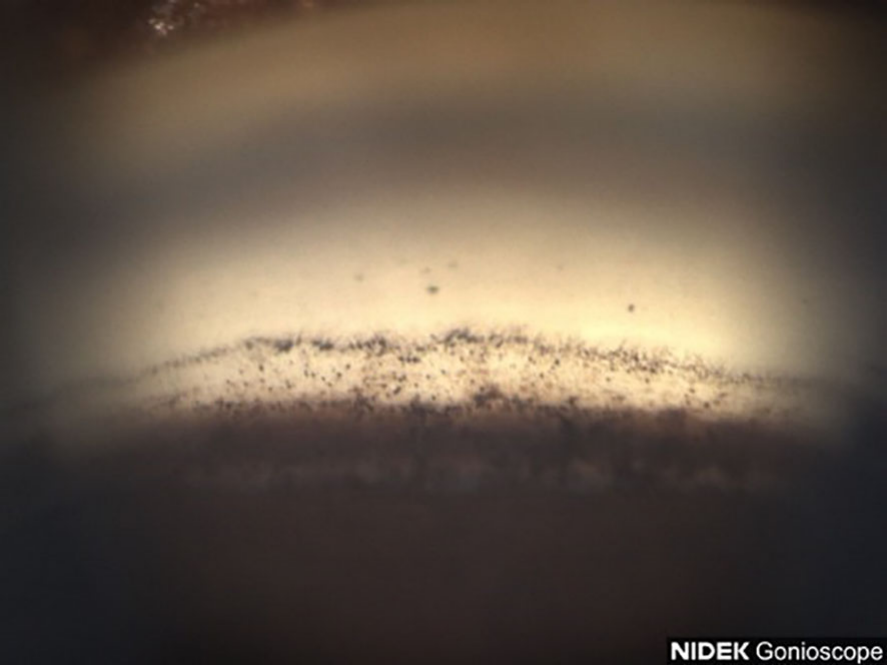
Case 5: Inferior angle (inverted by mirror).

The authors apologize for this error and state that this does not change the scientific conclusions of the article in any way.

